# Spectroscopic Ellipsometry Studies of *n*-*i*-*p* Hydrogenated Amorphous Silicon Based Photovoltaic Devices

**DOI:** 10.3390/ma9030128

**Published:** 2016-02-25

**Authors:** Laxmi Karki Gautam, Maxwell M. Junda, Hamna F. Haneef, Robert W. Collins, Nikolas J. Podraza

**Affiliations:** Wright Center for Photovoltaics Innovation & Commercialization and Department of Physics & Astronomy, University of Toledo, Toledo, OH 43606, USA; Laxmi.KarkiGautam@rockets.utoledo.edu (L.K.G.); Maxwell.Junda@rockets.utoledo.edu (M.M.J.); Hamna.Haneef@rockets.utoledo.edu (H.F.H.); Robert.Collins@utoledo.edu (R.W.C.)

**Keywords:** spectroscopic ellipsometry, hydrogenated silicon, infrared spectra, photovoltaic devices

## Abstract

Optimization of thin film photovoltaics (PV) relies on characterizing the optoelectronic and structural properties of each layer and correlating these properties with device performance. Growth evolution diagrams have been used to guide production of materials with good optoelectronic properties in the full hydrogenated amorphous silicon (a-Si:H) PV device configuration. The nucleation and evolution of crystallites forming from the amorphous phase were studied using *in situ* near-infrared to ultraviolet spectroscopic ellipsometry during growth of films prepared as a function of hydrogen to reactive gas flow ratio *R* = [H_2_]/[SiH_4_]. In conjunction with higher photon energy measurements, the presence and relative absorption strength of silicon-hydrogen infrared modes were measured by infrared extended ellipsometry measurements to gain insight into chemical bonding. Structural and optical models have been developed for the back reflector (BR) structure consisting of sputtered undoped zinc oxide (ZnO) on top of silver (Ag) coated glass substrates. Characterization of the free-carrier absorption properties in Ag and the ZnO + Ag interface as well as phonon modes in ZnO were also studied by spectroscopic ellipsometry. Measurements ranging from 0.04 to 5 eV were used to extract layer thicknesses, composition, and optical response in the form of complex dielectric function spectra (*ε* = *ε*_1_ + i*ε*_2_) for Ag, ZnO, the ZnO + Ag interface, and undoped a-Si:H layer in a substrate *n*-*i*-*p* a-Si:H based PV device structure.

## 1. Introduction

Development of thin film technologies based on amorphous silicon and germanium, including photovoltaic (PV) devices, involves understanding of material electrical and optical properties [1,2,3,4]. It is essential to measure, monitor, and control the thickness, structure, phase, and composition of solar cell component layers in the same configuration used in manufacturing, especially for devices processed over large areas. Doped (*n*-type and *p*-type) and undoped (intrinsic) hydrogenated silicon (Si:H) thin films are used in single, tandem, and multijunction solar cell applications in both *n*-*i*-*p* substrate and *p*-*i*-*n* superstrate configurations [5,6,7,8,9,10]. These Si:H films prepared by plasma enhanced chemical vapor deposition (PECVD) may exhibit several structural transitions during growth in the PV device configuration. The structural evolution of Si:H can be controlled by dilution of a reactive silicon carrying gases like silane (SiH_4_) with hydrogen (H_2_) during deposition. The microstructural evolution of Si:H has been studied for layers deposited on different bulk and thin film substrates with varying degrees of surface roughness, including native and thermal oxide coated crystalline silicon, glass, polyethylene naphthalate (PEN) polymer, as well as underlying structurally distinct Si:H layers prepared under different deposition conditions. A primary technique for studying this growth evolution is the use of near infrared (IR) to ultraviolet (UV) *in situ*, real time spectroscopic ellipsometry (RTSE) applied during Si:H thin film growth [2,4,7,11,12]. RTSE involves collection of ellipsometric spectra as a function of time during a process such as thin film deposition, typically using a multichannel instrument which collects all photon energies in parallel with serial readout of pixels arranged in a one dimensional (1-D) detector. The data acquisition time is typically chosen such that highest signal-to-noise is obtained via averaging of multiple optical cycles over a period of time in which typically only 0.1 to 10 Å of material accumulates. Koh *et al.* [7] reported the growth evolution of intrinsic Si:H on native oxide covered crystalline silicon, amorphous Si:H (a-Si:H) films prepared without additional hydrogen dilution, and on newly deposited ~200 Å p-type microcrystalline or nanocrystalline Si:H (nc-Si:H). These results demonstrate that the nature of the underlying material influences nucleation of crystallites, suppressing nucleation with underlying a-Si:H and promoting nucleation with underlying nc-Si:H. The growth evolution of *p*-layers on specular zinc oxide (ZnO) coated glass and the ability to promote a high nucleation density of nc-Si:H were studied by Rovira *et al.* [11]. In another study of the growth evolution of *p*-type Si:H on ZnO coated glass and ZnO over-coating tin oxide (SnO_2_), Koval *et al.* [12] reported that valid material properties and device performance correlations can be better realized for any given material when the properties are obtained from deposition on similar substrates with similar thicknesses as those used in the respective device. The generation of so-called “deposition phase diagrams” or “growth evolution diagrams” for vhf and rf PECVD of Si:H films determined that vhf PECVD shows significant differences in structural evolution with processing conditions, namely the plasma excitation frequency [13]. Growth evolution diagrams have been developed by Stoke *et al.* for intrinsic a-Si:H, amorphous silicon germanium alloys, and nc-Si:H for top, middle, and bottom cell *i*-layers used in triple junction devices [14]. Dahal *et al.* reported growth evolution diagrams for intrinsic and *p*-type Si:H deposited on unoptimized *n*-layer/ZnO/Ag back reflector (BR) coated PEN in *n*-*i*-*p* configuration PV devices [15]. Overall these results and analysis procedures developed here are applicable to more directly relating properties of layers in the device configuration, as obtained by non-destructive measurements, with variations in device performance. These types of measurements have been demonstrated for a-Si:H solar cells deposited on planar substrates as described here and also on those incorporating macroscopic roughness or texturing [16,17,18].

Both a-Si:H and nc-Si:H component materials are used in state-of-the-art Si:H based PV devices. nc-Si:H, either as individual layers or in PV junctions, has significant enhancement in near IR absorption of the solar spectrum and high stability under prolonged illumination in contrast to its amorphous counterpart [19,20]. The quantitative analysis, characterization, and control of the relative nanocrystalline and amorphous volume fractions within mixed-phase films is also a major challenge in Si:H manufacturing. Most often the nanocrystalline fraction is estimated from *x*-ray diffraction or Raman spectroscopy, which can yield values ranging an order of magnitude [6,21,22]. Although these measurements are valuable, limitations exist. Typically *ex situ* *x*-ray diffraction measurements average information over the full depth of a thin film sample, and *ex situ* Raman spectroscopy averages information over a finite penetration depth into the sample that is dependent upon the wavelength of the probing laser, its power, and the absorption coefficient of the material. Profiling these materials non-invasively is an even greater challenge due to probe penetration depth limitations and likely non-uniform crystallite fraction with depth into films. Si:H films may be inhomogeneous with thickness as crystallites nucleate from and coexist with the amorphous phase. Deconvolving gradients in crystallinity from *ex situ* *x*-ray diffraction and Raman spectroscopy measurements requires multiple samples, while *in situ* RTSE measurements applied during film deposition have been used to quantify structural gradients in crystallinity within a single film.

A wealth of information can be extracted from these types of RTSE measurements applied at a single spot on a sample surface, but additional property variations related to sample non-uniformity have also been obtained by *ex situ* mapping spectroscopic ellipsometry (SE) [23,24,25,26]. In mapping SE, the sample and multichannel ellipsometer, similar to the instrument used in RTSE studies, are mechanically translated with respect to the each other in one or more dimensions to obtain ellipsometric spectra as a function of spatial position. In the case of Si:H, simplified structural models based on results from RTSE measurements are applied to probe subtle variations in material opto-electronic response such as the band gap of a-Si:H, film thickness, surface roughness thickness, and nanocrystallite fraction in mixed phase materials. These types of measurements have been applied to Si:H [23,24], cadmium telluride [26], and copper indium gallium diselenide [25] PV devices ranging from tens of square centimeters on the laboratory scale to full industrially prepared panels. Thus, improvements in understanding and quantifying the structural transition of Si:H from amorphous to nanocrystalline, as obtained from single spot *in situ* RTSE measurements, can be applied to develop more advanced optical models and more thoroughly analyze mapping SE measurements collected over larger areas.

We have applied SE from 0.734 to 5.88 eV to extract layer thicknesses, interface composition, and optical response in the form of complex dielectric function spectra (*ε* = *ε*_1_ + i*ε*_2_) for all Ag, ZnO, and doped and undoped PECVD Si:H layers found in substrate *n*-*i*-*p* PV devices. These studies begin with characterization of ZnO/Ag BRs and have been applied over the near IR to UV spectral range [27,28]. The purpose of BR structures is to increase the optical path length of light within the absorber layer of the PV device, where each photon absorbed has the potential to generate electron-hole pairs and thus electrical current. Any light not absorbed in the first pass of light through the absorber layer is reflected or scattered by the BR back into the absorber layer. Thus PV absorber layers can be made thinner or from materials with low minority carrier diffusion lengths. Due to the substrate dependence of Si:H growth, the same ZnO/Ag BR structures were used to study the growth evolution of doped and undoped Si:H required for use in *n*-*i*-*p* a-Si:H PV devices. RTSE using a global Σσ-minimization analysis procedure has been used to track the behavior of structural transitions in Si:H deposited in the *n*-*i*-*p* PV device structure, as functions of hydrogen to reactive gas flow ratio *R* = [H_2_]/[SiH_4_], to produce growth evolution diagrams for undoped, *p*-type, and *n*-type layers. Global Σσ-minimization analysis of RTSE involves using test structural parameters, most commonly a bulk layer thickness (*d_b_*) and surface roughness thickness (*d_s_*), to numerically solve for test *ε* [29]. These test values of *ε* are then used to fit other ellipsometric spectra collected at different times when the film is relatively homogeneous. The approach is iterated in order to obtain numerically inverted *ε* yielding the lowest spectrally and time-averaged error, σ, over the multiple time measurements selected. The numerically inverted *ε* that minimizes σ are then used to determine structural parameter variations over the full set of RTSE data, with material transitions identified either in the structural parameters themselves or by increases in the error function. Virtual interface analysis (VIA) [30,31] has similarly been applied to track the depth profile of nc-Si:H as well as the formation and stabilization of voids throughout intrinsic layer nc-Si:H growth. In VIA, the full sample stack is not analyzed. Instead, optical properties of a pseudo-substrate are generated from ellipsometric spectra collected earlier in the deposition by numerically inverting the measurement to obtain *ε* using a simplified model consisting of a semi-infinite pseudo-substrate and a surface roughness layer. The effective *ε* for the pseudo-substrate contains information of all underlying material(s) in the sample stack and is then used as the semi-infinite substrate for analysis of subsequent data sets. In this sense, the time derivative of ellipsometric spectra is analyzed and full understanding of the underlying structure is not required in the analysis procedure. When combined with Σσ-minimization approaches for structurally graded Si:H, VIA yields *ε* for both the nc-Si:H and a-Si:H components as well as the time and bulk layer thickness dependence of component material fractions in the overlayer of material accumulated between each pseudo-substrate and subsequent data set pair. These techniques are used to provide guidance for the deposition and *in situ* characterization of a-Si:H, nc-Si:H, and mixed-phase (a+nc)-Si:H layers during growth in device structures.

In addition to RTSE studies of material growth evolution limited to the near IR to UV spectral range, we also have used *ex situ*, room temperature IR-extended SE (IR-SE) from 0.04 to 0.75 eV using a Fourier transform IR ellipsometer operating over this spectral range. This extension enables spectra in *ε* for Si:H and BR components layers to be determined from the mid-IR to UV wavelength range. Simultaneous analysis of ellipsometric spectra collected from multiple samples consisting of BR and *i*-layer/*n*-layer/BR stacks deposited on borosilicate glass substrates was used to yield a common *ε* for each layer while structural parameters such as *d_b_* and *d_s_* may be varied separately as in RTSE data analysis [4,7,11,13,14,15,27,28] and similar in methodology to the divided spectral range approach [32]. A common parameterization of *ε* is used to fit *ex situ* ellipsometric spectra collected from separate near IR-UV multichannel and FTIR ellipsometers. This approach yields a continuous set of spectra in *ε* for each material, although the particular beam spot location on the sample surface may not be the same during measurement using each ellipsometer [33]. The results of the analysis of IR-SE data were used to study absorption in the BR components, which can be used to extract electrical transport properties and phonon modes. In addition, *ε* for protocrystalline intrinsic a-Si:H extracted from IR-SE data is sensitive to the silicon-hydrogen bonding configuration. Comparison of optical absorption features affords a method of assessing film structural and chemical character, which then suggests ways to improve material quality and potentially device performance [34,35]. PV devices incorporating optimization principles based on IR spectroscopy and RTSE analyzed for each layer in the device configuration have exhibited relatively high performance [23,34].

*In situ* RTSE studies have been used to yield growth evolution phase diagrams of each doped and undoped Si:H layer in the *n-i-p* PV device configuration and structural evolution profiles of crystallite and void fractions. The information from growth evolution diagrams was used to design PV device structures, lacking the *p*-layer and incorporating only the amorphous phase, for characterization using *ex situ* IR-extended SE. Results of *ex situ* IR-extended SE combined with *ex situ* near IR-UV SE have been used to obtain spectra in *ε* from 0.04 to 5.0 eV for ZnO and a-Si:H in the BR/*n-i-p* a-Si:H solar cell configuration. Higher energy transitions related to electronic structure in Ag, ZnO, and a-Si:H; the band gap in ZnO and a-Si:H; a plasmon feature in the Ag + ZnO interface; IR vibrational modes related to chemical bonding in a-Si:H and ZnO; and free carrier absorption in Ag and the Ag + ZnO interface have been obtained from spectra in *ε*. In addition to information on each of these materials in the *n-i-p* device structure, the structural and optical properties derived here can be applied in the future analysis of *ex situ* SE in either single spot [36] or mapping configurations.

## 2. Experimental Details

Thin film doped and undoped Si:H films were deposited using a load-locked rf (13.56 MHz) PECVD reactor onto 6” × 6” borosilicate glass substrates coated with rf magnetron sputtered ZnO/Ag BRs as are commonly used in *n-i-p* configuration solar cells. Si:H *n*-, *p-*, and *i-*layers have been prepared using different hydrogen dilution ratios, *R*, onto BR coated substrates or those otherwise mimicking the outermost previous layer in the device structure to generate growth evolution diagrams and identify the optimum conditions for protocrystalline a-Si:H for solar cells. Table 1 lists the deposition parameters used for fabrication of each layer including gas flows, pressure (*p*), power density (*P*), and substrate temperature (*T*). The deposition conditions used here were adopted from the Dahal *et al.*, 2013 and Dahal *et al.*, 2014 [23,37]. The deposition was done in the same chamber corresponding to reasonable device quality materials as evidenced by incorporation in *n*-*i*-*p* solar cells without textured BRs yielding ~7.5% efficiency. Maximum device performance parameters are open circuit voltage of 0.90 V, short circuit current of 12.5 mA/cm^2^, and fill factor of 70%. The open circuit voltage and fill factor are reasonable for moderate quality *n*-*i*-*p* solar cells. The short circuit current is low as the specular BR does not produce the level of scattering expected from a textured BR where the optical path length of long wavelength light not initially absorbed in the intrinsic a-Si:H layer is increased. This increase in optical path length results in increased current density, which is absent for specular devices. The *n*-type Si:H films were deposited onto BR coated borosilicate glass. The intrinsic layers were deposited onto BR’s coated with *n*-type a-Si:H prepared at *R* = 50. The *p-*type Si:H films were deposited onto borosilicate glass initially coated with ~3000 Å thick intrinsic a-Si:H prepared at *R* = 10. This intrinsic layer is deposited to eliminate any contributions to the microstructural evolution from the underlying glass substrate. Variable parameters were *R =* [H_2_]/[SiH_4_] for all three layers. The dopant gas ratios for *n-* (*D =* [PH_3_]/[SiH_4_]) and *p-*layers (*D =* [B_2_H_6_]/[SiH_4_]), which can have significant influence on structural and the electronic properties, were fixed at *D* = 0.0125. Cr, Ag, and ZnO layers were prepared by rf magnetron sputtering at room temperature. Here, Cr was used as an adhesion interlayer to avoid delamination of the Ag film from the borosilicate glass. The ZnO/Ag BR structures were prepared under identical conditions for each sample to study how the Si:H layers grow in the device configuration.

RTSE was performed *in situ* at a single spot during deposition using a rotating-compensator multichannel ellipsometer (J. A. Woollam Company model M-2000) that can measure ellipsometric spectra (in the form of *N* = cos 2ψ, *C* = sin 2ψ cos Δ, S = sin 2ψ sin Δ) from 0.734 to 5.88 eV with a minimum data acquisition time of 50 ms [38,39]. This type of instrument collects ellipsometric spectra at all photon energies in parallel by a combination of a 1-D linear detector array and serial pixel readout. Dual detectors are required to access this spectral range, and consist of a silicon based charged coupled device (CCD) and indium gallium arsenide photodiode array (PDA). RTSE measurements were collected at the respective deposition temperature at angles of incidence near 70° and spectra obtained from single optical cycles were averaged over 1.5 s intervals to increase the signal-to-noise ratio. Analysis of experimentally collected RTSE data was performed using J. A. Woollam Co. CompleteEASE software (Lincoln, NE, USA). The time evolution of *d_b_* and *d_s_* as well as the spectroscopic *ε* of the bulk Si:H layers were extracted from RTSE data using a global Σσ-minimization procedure. For Si:H films, global Σσ-minimization analysis of RTSE involves using test *d_b_* and *d_s_* values for the Si:H layer being deposited on top of a pre-defined substrate stack to numerically solve for test *ε* of the Si:H layer [29]. The test values of *ε* are then used to fit other spectra collected at different times when the film is relatively homogeneous, typically near 100–200 Å in accumulated material thickness for Si:H films where structural transitions have not yet had time to mature. The approach is applied in the regime prior to crystallite nucleation and is iterated in order to obtain numerically inverted *ε* yielding the lowest spectrally and time-averaged error, σ, over the multiple time measurements selected. The numerically inverted *ε* minimizing σ are taken to be the best representation of the a-Si:H optical properties and are then used to determine structural parameter variations over the full set of RTSE data, with the nucleation of crystallites from the a-Si:H matrix identified by a sharp increase in the surface roughness thickness (≥1 Å between successive time points) and by increases in σ as spectra in *ε* for a-Si:H are no longer adequate to fit ellipsometric spectra collected as nanocrystallites evolve. The unweighted error function, σ, is defined by [40]:

(1) $$\sigma =\sqrt{\frac{1}{3N-M}\sum_{j=1}^{N}\left[\begin{array}{c} {\left(\cos2\psi_{j}^{\mathrm{mod}}-\cos2\psi_{j}^{\exp}\right)}^{2}\\ +{\left(\sin2\psi_{j}^{\mathrm{mod}}\cos\Delta_{j}^{\mathrm{mod}}-\sin2\psi_{j}^{\exp}\cos\Delta_{j}^{\exp}\right)}^{2}\\ +{\left(\sin2\psi_{j}^{\mathrm{mod}}\sin\Delta_{j}^{\mathrm{mod}}-\sin2\psi_{j}^{\exp}\sin\Delta_{j}^{\exp}\right)}^{2} \end{array}\right]}$$

where *N* is the number of measured values; and *M* the number of fit parameters; “*exp*” denotes experimental spectra; and “*mod*” denotes that generated from the model. An advantage of conducting *in situ* RTSE measurements during growth of Si:H by PECVD is that spectra in *ε* can be obtained prior to the exposure of the sample to ambient and potential oxidation.

Near IR to near UV room temperature ellipsometric spectra over a range from 0.734 to 5.88 eV were collected at a single spot *ex situ* prior to the collection of the *ex situ* IR-SE data as those measurements were not able to be collected *in situ* during film growth. IR-SE data was collected at a single spot using a similar single rotating compensator instrument (J. A. Woollam Company, Lincoln, NE, USA, model FTIR-VASE) from 0.04 to 0.75 eV at 1 cm^−1^ resolution [41]. The angle of incidence for all *ex situ* measurements was nominally 70°. Ellipsometric spectra over the mid-IR to near UV range collected from the two instruments were analyzed simultaneously using a common parameterization for *ε* based on structural models initially developed from RTSE and near IR to near UV measurements and J. A. Woollam Co. WVASE software (Lincoln, NE, USA). The error function in Equation 1 is also used for analysis and fitting of *ex situ* IR-SE data.

For *in situ* RTSE, *ex situ* near IR to UV SE, and *ex situ* IR-extended SE data analysis, the optical response of the surface roughness layer of thickness *d_s_* for Si:H and ZnO is represented using Bruggeman effective [42,43] medium approximation mathematically represented as:

(2) $$\sum_{n}f_{n}\frac{\varepsilon_{n}-\varepsilon }{\varepsilon_{n}+2\varepsilon }=0.$$

in this expression, material fractions (*f_n_*) and component material optical response (*ε_n_*) are used to generate a composite *ε* for the mixture. For surface roughness in this work, spectra in *ε* from Bruggeman effective medium approximation consist of 0.5 void and 0.5 underlying material volume fractions, regardless of composition of the underlying layer.

The structural model constructed for each sample consists of a stratified layer stack of optically distinct materials, which may be continuous films, interfaces with unique *ε* and thickness, or Bruggeman effective medium approximation layers. Ellipsometric spectra are modeled using a scattering matrix formalism [44] in which 2 × 2 matrices based on Fresnel coefficients and wave propagation of light through media is generated for the semi-infinite substrate and ambient, each layer of finite thickness, and the interfaces between each optically distinct layer. Matrices are calculated for the components of the incident electric field both parallel and perpendicular to the plane of incidence. In general, ellipsometric spectra are sensitive to spectra in *ε* for each material, including the ambient and substrate, and the thicknesses of optically finite layers.

## 3. Results and Discussion

### 3.1. Optical Characterization of Back Reflector Components and Structure

The first layers deposited for *n*-*i*-*p* configuration a-Si:H solar cells comprise the ZnO/Ag BR structure. Therefore, ellipsometric spectra from 0.04 to 5.0 eV are collected and analyzed for a ZnO/Ag BR structure. The models and thicknesses described here first correspond to a ZnO coated Ag BR sample, while variations in properties due to growth of over-deposited *n*- and *i*-type a-Si:H layers is described in Section 3.3.1. All layers were deposited without vacuum break with conditions given in Table 1. *In situ* SE data from 0.734 eV to 5.88 eV was collected for each deposited layer and the model generated was used for extended spectral range IR-SE analysis.

#### 3.1.1. Ag and ZnO + Ag Interface Properties

**Figure 1:**
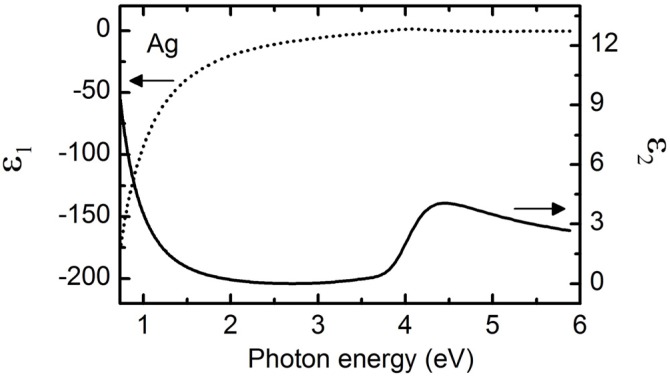
Complex dielectric function spectra, *ε* = *ε*_1_ + i*ε*_2_, (arrow pointing left for *ε*_1_ axis, arrow pointing right for *ε*_2_ axis) from 0.734 to 5.88 eV for a semi-infinite Ag film parameterized with a combination of a Drude oscillator and two oscillators assuming critical point parabolic bands (CPPB) with parameters listed in Table 2.

Data collected for semi-infinite Ag substrate were taken before ZnO layer deposition at room temperature and were analyzed in the energy range from 0.734 to 5.88 eV. Figure 1 shows spectra in *ε* for Ag parameterized by a combination of a Drude oscillator [45], a higher energy transition assuming critical point parabolic bands (CPPB) [46], and a constant additive term to *ε*_1_ denoted as *ε*_∞_. The surface roughness is represented by two Lorentz oscillators [47] with *ε*_∞_ = 1. The Drude oscillator is represented by:

(3) $$\varepsilon (E)=\frac{-\hbar^{2}}{\varepsilon_{0}\rho (\tau E^{2}+i\hbar E)}$$

where *ħ* is the reduced Planck’s constant, *ε*_0_ is the permittivity of free space, *τ* is the scattering time, and *ρ* is the resistivity. Each CPPB oscillator is represented by:

(4) $$\varepsilon (E)=Ae^{i\phi }{\left\{\frac{\Gamma }{\left[{2E}_{n}-2E-i\Gamma \right]}\right\}}^{\mu }$$

where *A*, *E_n_*, *Γ*, *μ*, and *φ* are the amplitude, resonance energy, broadening, exponent, and phase of the critical point, respectively. The exponent *μ* can assume the values of 1/2, 0, and −1/2 depending on whether the critical points are one, two, or three dimensional in nature. In this work, only the one dimensional CPPB oscillator has been used, and so its value was fixed at *μ* = 0.5. Each Lorentz oscillator is represented by:

(5) $$\varepsilon (E)=\frac{A\Gamma E_{0}}{\left[{E_{0}}^{2}-E^{2}-i\Gamma E\right]}$$

where *A*, *Γ*, and *E_0_* represent amplitude, broadening, and resonance energy respectively. All parameters describing Ag and its surface roughness are listed in Table 2. A resistivity of 3.02 ± 0.03 × 10^−6^ Ωcm and a scattering time of 16.7 ± 0.1 fs were determined from the Drude oscillator parameters of the Ag film.

**Figure 2:**
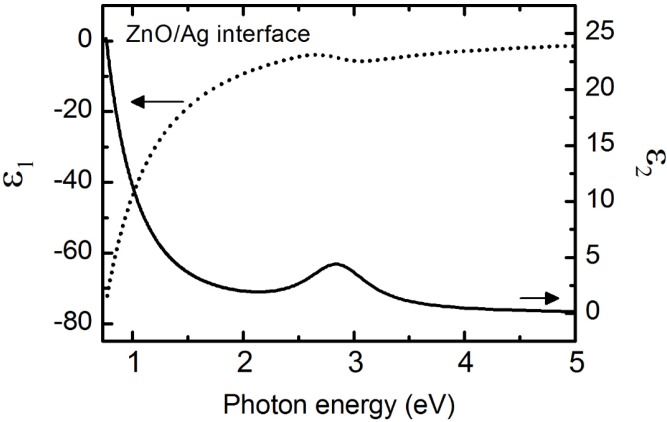
Spectra in *ε* (arrow pointing left for *ε*_1_ axis, arrow pointing right for *ε*_2_ axis) from 0.734 to 5.0 eV for the 108 ± 1 Å thick ZnO + Ag interface layer parameterized with a Lorentz and a Drude oscillator with parameters listed in Table 3.

The structural model for the ZnO/Ag BR in the energy range 0.734 to 5 eV consisted of a semi-infinite Ag metal layer deposited onto glass, a 108 ± 10 Å ZnO + Ag interfacial layer, a 3059 ± 3 Å bulk ZnO layer, and a 80 ± 1 Å surface roughness represented using Bruggeman effective medium approximation of 0.5 ZnO and 0.5 void volume fractions. Parametric expressions were used to describe *ε* for Ag, ZnO, and the ZnO + Ag interface and are listed in Table 2 and Table 3. Previous studies of ZnO/Ag interfaces in the BR of thin film *n*-*i*-*p* a-Si:H PV shows that the optically determined value of Ag surface roughness obtained from RTSE is very close to that measured with atomic force microscope (AFM) with *d_s,RTSE_* (Å) = 0.96 *d_s,AFM_* (Å) + 5 Å [28]. The *d_s,RTSE_* = 30 ± 2 Å for Ag corresponds to a *d_s,AFM_* = 26 Å. After deposition of ZnO, the ZnO/Ag interface layer thickness is reported by Dahal *et al.* as *d_i_* (Å) = 1.98 *d_s_* (Å) + 17.5 Å. The interface layer thickness predicted from the Ag surface roughness in this work is 76.9 Å as compared to that obtained in our parametric analysis of 108 Å [28]. Our parametric value slightly overestimates the prediction, however in Dahal *et al.* [28] the samples with similar Ag surface roughness, 25–30 Å, also has an interface thickness of 75–110 Å which are greater than the linear prediction. Figure 2 shows that the spectra in *ε* obtained for the ZnO + Ag interface is optically different than Ag and ZnO alone and can be modeled by a Lorentz oscillator and a Drude oscillator in the near IR to near UV range (0.734 to 5 eV) with *ε*_∞_ = 1. The ZnO + Ag interface exhibits a clear localized particle plasmon absorption feature which can be modeled using a Lorentz oscillator with a resonance energy at 2.83 ± 0.01 eV [27,48]. A resistivity of 3.7 ± 0.5 × 10^−5^ Ωcm and a scattering time of 2.7 ± 0.3 were determined from the Drude oscillator parameters of the ZnO + Ag interface. These values indicate that when compared to bulk Ag, the interface is less conductive due to incorporation of higher resistivity undoped ZnO and potentially more disordered as suggested by the lower scattering time. Over this spectral range, *ε* for ZnO was initially fit using two CPPB oscillators, *ε*_∞_, and a zero-broadened Sellmeier oscillator [49] represented by:

(6) $$\varepsilon (E)=\frac{A}{\left(E_{n}^{2}-E^{2}\right)}$$

where *A* and *E_n_* represent the amplitude and resonance energy, respectively.

#### 3.1.2. Phonon Modes in ZnO

**Figure 3:**
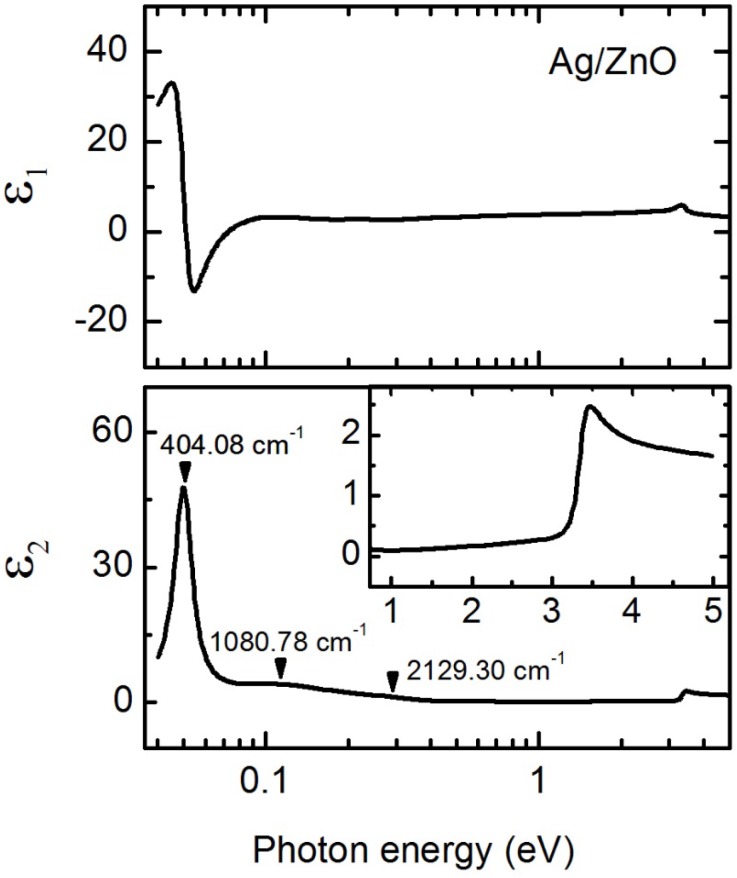
Spectra in *ε* (top panel, real part *ε*_1_; bottom panel, imaginary part *ε*_2_) from 0.04 to 5.0 eV for a 3010 ± 2 Å thick ZnO film on Ag, with *ε* for ZnO parameterized using a combination of two CPPB and three Lorentz oscillators with parameters listed in Table 4. The inset shows high-energy electronic transitions in *ε*_2_.

The analysis was extended to the IR by fitting parameters defining *ε* for ZnO only and fixing those defining *ε* for Ag and the ZnO + Ag interface as well as the interface layer thickness. This analysis approach was chosen because free carrier absorption represented by the Drude feature dominates the IR response of Ag and the ZnO + Ag interface layers and is already established from near IR to UV spectral range analysis. A common parameterization of *ε* for the ZnO was applied for the data collected from the two instruments with spectral ranges from 0.04 to 0.734 eV and 0.734 to 5.0 eV, respectively, although the bulk ZnO layer thickness was allowed to vary for the ellipsometric spectra collected from each respective instrument to account for measurement on different spots over the sample surface. A common surface roughness thickness between the two sets of measured spectra was obtained, as this effect will vary less with non-uniformity than the overall bulk layer thickness. Figure 3 shows *ε* for ZnO represented by a combination of CPPB oscillators for electronic transitions, Lorentz oscillators representing IR phonon modes, and a constant real additive term *ε*_∞_ to account for dispersion from absorption features outside the measured spectral range from 0.04 to 5 eV with parameters given in Table 4. The near IR to near UV range shows only small absorption below the lowest direct transition at 3.364 eV as expected for direct band gap ZnO [50]. Phonon modes for wurtzite ZnO are Γ_opt_ = 1A_1_ + 2B_1_ + 1E_1_ + 2E_2_, with A_1_ and E_1_ modes IR-active. Only one characteristic transverse optical (TO) mode for ZnO with E_1_ symmetry at 0.0501 eV (404.08 cm^−1^) is resolved for this sample [51,52,53]. Weak absorption bands in the spectral region from 0.134 to 0.264 eV (1080 to 2130 cm^−1^) have been observed and are often associated with hydrogen*-*associated bending modes; stretching modes of hydrogen bonded to heavier elements like zinc; and various carbon, oxygen, and nitrogen*-*related stretching modes not involving hydrogen [54]. These types of peaks are analogous to those found in the absorbance spectra from traditional unpolarized FTIR measurements, which lack sensitivity to discerning thickness and the full complex optical properties simultaneously—a capability of SE measurements.

### 3.2. RTSE Monitoring of Si:H in n-i-p Solar Cell Devices

The films used to develop growth evolution diagrams for doped and undoped Si:H deposited in the glass substrate/BR/*n*-*i*-*p* a-Si:H device configuration were grown as a function of *R* in an effort to probe the subtle fluctuations expected as the material transitions from amorphous to nanocrystalline [31,55]. A distinct type of roughening transition is reported in which crystallites nucleate from the growing amorphous phase. Because of the low crystallite nucleation density as observed by Fujiwara *et al.* and Ferlauto *et al.* [4,31], the growth of crystalline protrusions produce a roughness layer that increases promptly when compared to increases in bulk layer thickness. Thus, the onset of roughening identifies a transition to mixed-phase amorphous+nanocrystalline (a+nc)-Si:H film growth accompanied by changes in the film optical properties. This behavior denotes the amorphous-to-mixed-phase [a→(a+nc)] transition. Simple roughening of the amorphous phase also tends to exhibit a lower increase in *d_s_*, accompanied by only minimal increases in σ, with accumulated bulk layer thickness. Crystallites nucleating from the amorphous phase grow preferentially over the surrounding material, until the point at which the crystallites cover the surface. The disappearance of the amorphous phase and coalescence of crystallites is denoted as the mixed-phase-to-single-phase nanocrystalline [(a+nc)→nc] transition. The a→(a+nc) and (a+nc)→nc transitions were detected using RTSE monitoring and data analysis in this work. The Si:H films prepared at low *R* remain in the amorphous growth regime throughout the deposited thickness. VIA was applied to RTSE data collected during growth for Si:H transitioning from amorphous to nanocrystalline [31,56,57].

**Figure 4:**
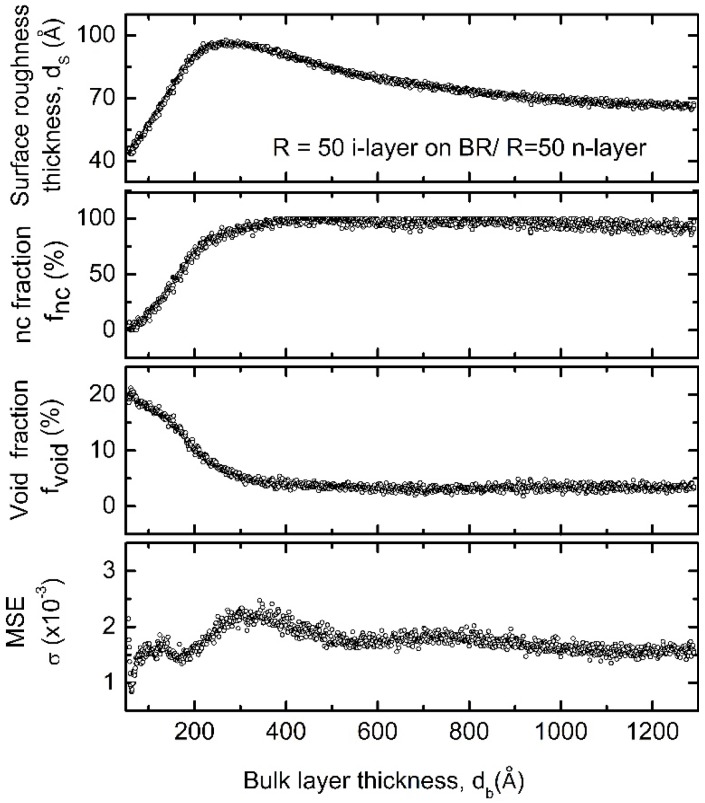
Mean square error (MSE), void fraction (*f_void_*), nanocrystalline volume fraction (*f_nc_*), and surface roughness thickness (*d_s_*) in the top ~10 Å of the bulk layer, plotted *versus* the accumulated bulk layer thickness for an intrinsic hydrogen diluted *R* = [H_2_]/[SiH_4_] = 50 Si:H film deposited on a 200 Å *R* = 50 *n*-type a-Si:H over-deposited onto a ZnO/Ag back reflector (BR), as determined by virtual interface analysis (VIA) applied to real time spectroscopic ellipsometry (RTSE) data. Spectrally averaged mean error for *f_void_*, *f_nc_*, and *d_s_* are 0.3%, 2.4%, and 0.8 Å respectively.

**Figure 5:**
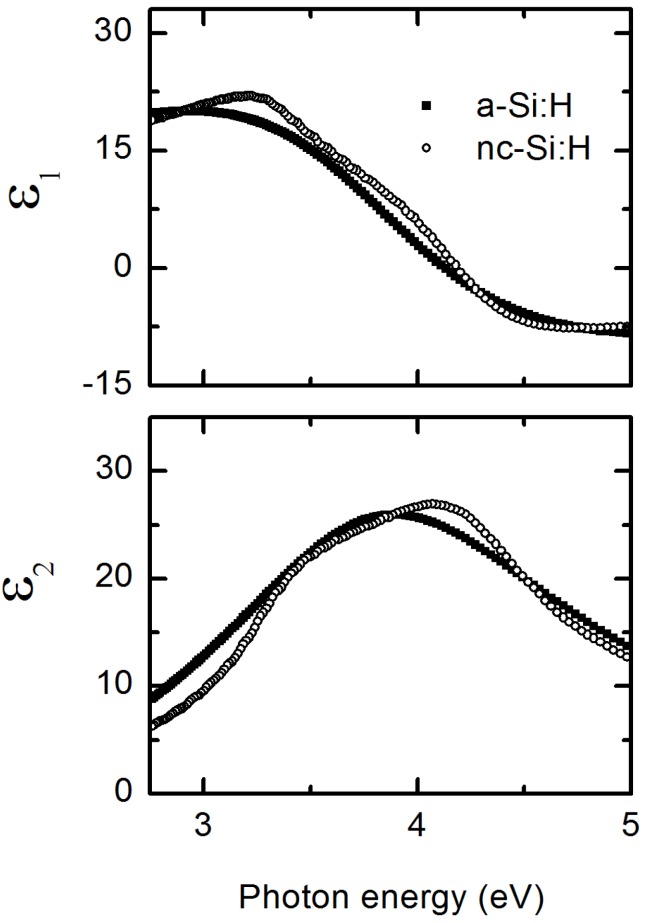
Spectra in *ε* (top panel, real part *ε*_1_; bottom panel, imaginary part *ε*_2_) of a-Si:H and nc-Si:H reference material used in VIA applied over a spectral range from 2.75 to 5.0 eV. Spectra in *ε* for a-Si:H and nc-Si:H were obtained from analysis of RTSE data and by numerical inversion at a bulk layer thickness of 200 and 1150 Å, respectively.

Figure 4 shows an example of the results of VIA applied to RTSE data to obtain the surface roughness thickness, nanocrystallite fraction, void fraction, and average mean square error (Equation (1)) as functions of the bulk layer thickness for a *R* = 50 *i*-layer on a BR over-coated with a 200 Å *R* = 50 *n*-layer. The VIA applied here utilizes spectra from 2.75 to 5.0 eV and *ε* for a-Si:H and nc-Si:H components as shown in Figure 5. Spectra in *ε* for nc-Si:H was obtained from the end of the respective deposition when the film is known to be fully nanocrystalline, ~1150 Å of a 1300 Å thick film using the same optical model as was used for the *i*-layer growth evolution diagram. In this model the free parameters are *d_b_* and *d_s_*. Spectra in *ε* for the amorphous phase was taken from the analysis of *R* = 15 deposition corresponding to a time within the first ~200 Å of bulk material prior to the nucleation of nanocrystallites. There is strong optical contrast between the two sets of *ε* for Si:H, in that the amorphous phase has only a single broad resonance while that of nanocrystallite material has two features representative of dampened and broadened critical point features found in single crystal silicon [58]. These reference spectra in *ε* for a-Si:H and nc-Si:H, along with that for void (*ε* = 1), were then used in a three component Bruggeman effective medium approximation [42,43] layer and a least-squares regression within the VIA with *d_s_* and the relative nanocrystallite (*f_nc_*) and void (*f_void_*) fractions as free parameters and the amorphous fraction constrained (*f_a_* = 1 − *f_nc_* − *f_void_*).

Results of VIA show an increase in surface roughness followed by a decrease within the first ~300 Å of material accumulation, indicating crystallite nucleation on the substrate followed by coalesce of the clusters. The nanocrystallite fraction increases with bulk layer thickness, then converges to 1.0 as expected for a nanocrystalline film. Voids initially appear with the nucleation of crystallites, which then subsequently decrease and stabilize near *f_void_* = 0.04 throughout the growth of this layer. Depending on the source of reference *ε* for nc-Si:H, this behavior could indicate that the grains under these conditions were not well passivated with a-Si:H as is desirable in nc-Si:H PV [22,59]. Optimized nanocrystalline/microcrystalline PV devices often incorporate layers prepared at lowest hydrogen dilution where crystallite growth can occur, and nc-Si:H layers are often fabricated using hydrogen dilution grading approaches to manipulate the degree of crystallinity. For very high values of hydrogen dilution, such as *R* = 50 in this example, the material is likely not optimized for solar cells, because cracks related to voids can promote shunts in the cells and channels by which contamination (e.g., oxygen) can enter into the layer [14,21,60].

Comparison of the structural behavior of the a→(a+nc) and (a+nc)→nc transitions as a function of single deposition parameters has been used to produce so-called deposition phase diagrams or growth evolution diagrams which have helped guide the development of optimization principles in Si:H based PV. For example, the structural evolution can be controlled by the dilution of reactive silicon carrying gases with hydrogen during the deposition process. Films prepared at low *R* remain amorphous throughout their total thickness, while those prepared at higher *R* nucleate crystallites. The thickness at which the a→(a+nc) transition occurs decreases with increasing *R*. Optimum a-Si:H based PV devices incorporate layers prepared at the highest *R* that will remain amorphous throughout the full thickness of the absorber layer while optimum nc-Si:H PV incorporates layers prepared at the lowest *R* where crystallite growth can occur [7,59,60,61,62]. For the case of a-Si:H, the additional hydrogen dilution improves ordering in the a-Si:H network, while for nc-Si:H low hydrogen dilution ensures that hydrogen etching does not occur and the grain boundaries remain well-passivated.

**Figure 6:**
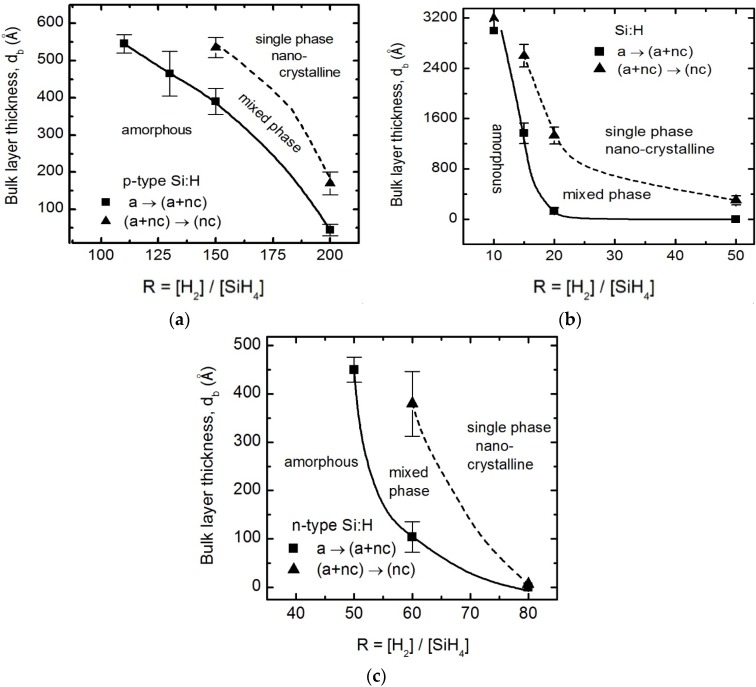
Growth evolution diagrams obtained from analysis of RTSE data for (**a**) *p*-type; (**b**) intrinsic; and (**c**) *n*-type Si:H as a function of variable hydrogen dilution *R* = [H_2_]/[SiH_4_] in the *n*-*i*-*p* solar cell device structure. The data values and connecting lines depict the a→(a+nc) and (a+nc)→nc structural transitions of doped and undoped Si:H prepared at conditions described in Table 1. Arrows pointing upward indicate the respective transition occurs beyond the maximum thickness measured.

The growth evolution diagrams of *n*-type, intrinsic, and *p*-type Si:H layers in the *n*-*i*-*p/*BR/glass configuration are depicted in Figure 6. The *n*-type Si:H layers are prepared at *T* = 200°C, *p* = 1.5 Torr, *P* = 0.032 W/cm^2^, and *D* = 0.0125 as a function of *R* varied from 20 to 80. For *R* < 50 the *n*-layer remains amorphous at least to a thickness of 500 Å. At *R* = 50 nanocrystallites nucleate in the *n*-type Si:H at about 450 Å of bulk layer thickness. The amorphous material prior to the a→(a+nc) transition of these depositions is protocrystalline [2]. A ~200 Å thick *n*-layer is typical for *n*-*i*-*p* configuration devices, and the best *R* for optimized *n*-*i*-*p* a-Si:H solar cells with a protocrystalline *n*-layer is identified here as near *R* = 50. As *R* is further increased, nanocrystallites nucleate within the amorphous phase at decreasingly lower thicknesses as indicated by the a→(a+nc) transition thicknesses. Films nucleating crystallites and grown to sufficient thickness show the (a+nc)→nc transition with crystallites coalescing at similarly decreasing thickness with increasing *R*. The film at *R* = 60 nucleates crystallites at ~100 Å and coalescence occurs at ~380 Å. These transitions occur much sooner for *R* = 80 leading to nanocrystallite formation in the very beginning of the deposition, making it unsuitable for an optimum *n*-type layer in single junction a-Si:H devices.

In both *n*-*i*-*p* substrate and the *p*-*i*-*n* superstrate PV device configurations, most incident photons are absorbed in the intrinsic layer with photo-generated electrons and holes transported to the contacts. Hence, optimization of *i*-layer is critical and the optical response and phase composition of these intrinsic layers tremendously impact solar cell performance. The intrinsic Si:H layers are prepared at *T* = 200 °C, *p* = 0.8 Torr, and *P* = 0.04 W/cm^2^ as a function of *R* varied from 10 to 50. The growth evolution diagram for intrinsic Si:H as a function of variable hydrogen dilution, 10 ≤ *R* ≤ 50, onto *n*-layer coated BRs has been developed and is shown in Figure 6. The hydrogen dilution and thickness of *n*-layer was fixed at *R* = 50 and ~200 Å, based on protocrystallinity observed in the *n*-layer growth evolution diagram. For the intrinsic layer, *R* = 15 is the lowest hydrogen dilution ratio at which the a→(a+nc) transition is observed within ~3000 Å of layer growth. The decrease in the (a+nc)→nc thickness with *R* may indicate higher nucleation density of crystallites for higher hydrogen dilution. Hence, *R* = 10 is identified here as optimized for *n*-*i*-*p* a-Si:H solar cells incorporating a ~3000 Å thick protocrystalline absorber [2].

The thickness of the *p*-layer should be thin enough to maximize transparency but thick enough to generate an electric field in the intrinsic layer. Typical *p*-layer thicknesses are ~100–150 Å, and a large optical band gap assists in minimizing parasitic absorption of incident light within this layer. Within the amorphous and protocrystalline phase the band gap of the *p*-layer generally increases with increasing *R*. The intrinsic layer, *p*-layer, and their interface are most directly responsible for open circuit voltage optimization, which can be guided using growth evolution diagrams [63,64]. The *p*-type Si:H layers are prepared at *T* = 100°C, *p* = 1.5 Torr, *P* = 0.066 W/cm^2^, and *D* = 0.0125 as a function of *R* varied from 50 to 200 on borosilicate glass initially coated with ~3000 Å thick intrinsic a-Si:H prepared at *R* = 10. From the growth evolution diagram, it can be observed that the *p*-layer depositions with *R* > 150 nucleate crystallites within the typical *p*-layer thickness used in a-Si:H based PV. The *R* = 110 film grows initially as a-Si:H and the a→(a+nc) transition occurs after a bulk layer thickness of 545 Å. Depositions at 50 ≤ *R* ≤ 100 indicate that this transition occurs for thicknesses greater than the deposited 650 Å, which is outside the range of interest for solar cells. At *R* = 200, the a→(a+nc) transition occurs at a bulk thickness of 40 Å, and the (a+nc)→nc transition occurs within 200 Å. The *p*-layer should be deposited at the maximum *R* that can be sustained without crossing the a→(a+nc) transition boundary throughout the desired thickness of 100–150 Å here. This *p*-layer growth evolution diagram is comparable to previously published diagrams [11,12,63,64].

**Figure 7:**
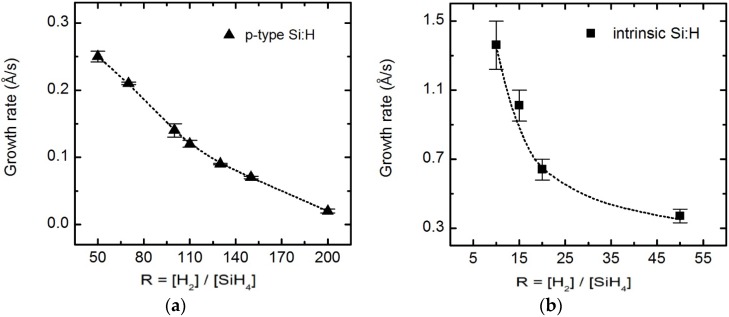

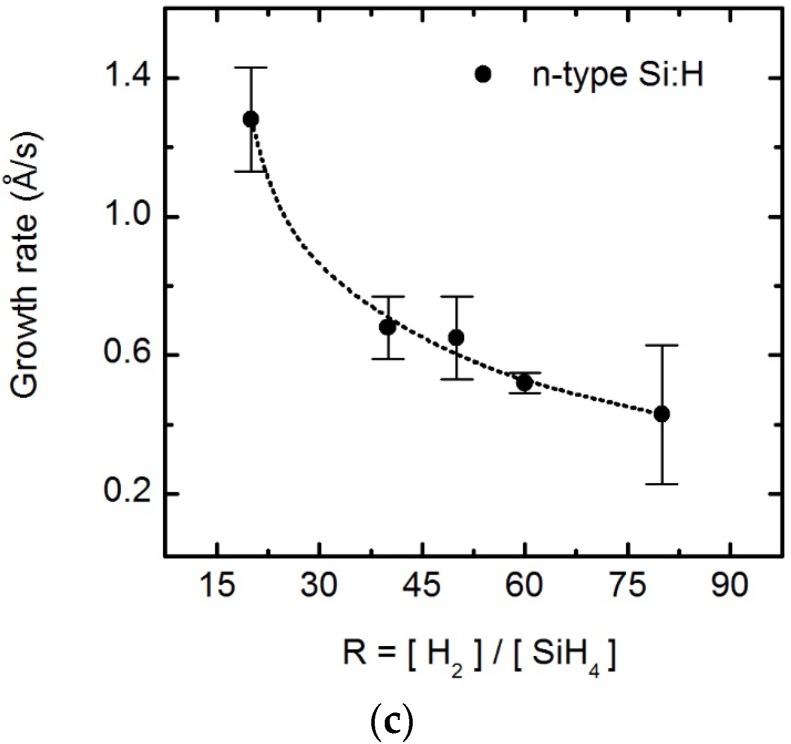
Deposition rates of (**a**) *n*-, (**b**) *i-*, and (**c**) *p-*layers on ZnO/Ag, *n-*layer/ZnO/Ag, and *i-*layer/glass, respectively, as functions of *R*.

**Figure 8:**
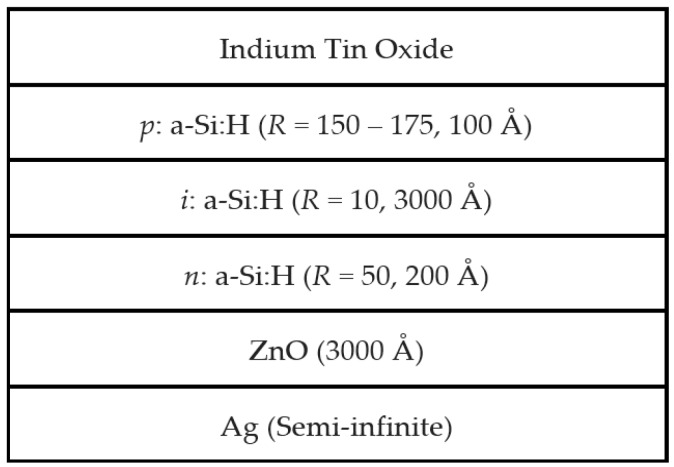
Schematic of a single junction a-Si:H based solar cell prepared in the *n*-*i*-*p* configuration. Each amorphous or protocrystalline Si:H layer is optimized to a value of *R* with an intended thickness.

The slope of *d_b_*, r(t) = d(*d_b_*(t))/dt, was used to determine the deposition rate of each film even though *ε* for films containing nanocrystallites are not accurate due to phase evolution with thickness. Figure 7 shows variations in growth rate as functions of *R* for *n*-, *i*-, and *p*-layers. The deposition rate shows a familiar trend in that it decreases with increasing *R*. Increased atomic hydrogen present in the plasma resulting from the increase in hydrogen dilution may etch weakly bonded material, leading to the removal of potentially defect-rich material and slowing the deposition rate. These deposition rates were later used in VIA of RTSE data collected for films nucleating crystallites. A schematic diagram showing a single junction *n*-*i*-*p* device with *R* optimized for the intended thicknesses of each a-Si:H layer is shown in Figure 8.

### 3.3. Ex Situ SE Study of a-Si:H in n-i-p Configuration Solar Cells from the Mid-IR to Near UV

Ellipsometric spectra from 0.04 to 5.0 eV were collected and analyzed for a ZnO/Ag BR over-coated with intrinsic a-Si:H and *n*-type a-Si:H layers. This ZnO/Ag BR sample was over-coated with a-Si:H to determine *ε* for a-Si:H over the 0.04 to 5.0 eV range as well as identify modifications to the underlying ZnO due to this over-deposition. The structural model for the a-Si:H coated ZnO/Ag BR consisted of a semi-infinite opaque Ag metal layer, a 108 Å ZnO + Ag interfacial layer with fixed thickness from the previous analysis given in Table 3, an average 2751 ± 5 Å bulk ZnO layer produced by the mean *d_b_* values obtained from the simultaneous fitting of the IR and the near IR-UV range spectra, a 84 ± 1 Å 0.5 *n*-type a-Si:H + 0.5 ZnO Bruggeman effective medium approximation interfacial layer, a 278 ± 1 Å a-Si:H *n*-layer, a 30 ± 1 Å 0.5 intrinsic + 0.5 *n*-type a-Si:H Bruggeman effective medium approximation interfacial layer, a 3621 ± 2 Å bulk intrinsic a-Si:H layer, and a 29 ± 1 Å surface roughness represented using Bruggeman effective medium approximation of 0.5 intrinsic a-Si:H/0.5 void volume fractions. The *n-*layer + ZnO interface, *n-*layer bulk layer, and *n-*layer surface roughness thicknesses are obtained from *in situ* RTSE measurements and analysis prior to intrinsic a-Si:H deposition. The intrinsic + *n-*type a-Si:H interface thickness is set at the same value as the *n-*layer surface roughness assuming that over-deposited intrinsic a-Si:H fill the voids in the *n-*layer surface. Parameters describing *ε* for ZnO and a-Si:H are listed in Table 5. As with the IR extended analysis of the ZnO/Ag sample, a common parameterization of *ε* for the materials over the full spectral range was applied, the bulk layer thicknesses for the ZnO and intrinsic a-Si:H layers were fit independently for spectra collected from each instrument, and all other layer thicknesses were either fixed from prior analyses or kept common between the two sets of spectra. For the *i*-layer, the nominal substrate temperature and hydrogen dilution ratio were *T* = 200°C and *R* = 10, respectively. The optimized *n*-type a-Si:H thickness was fixed at 278 Å for *R* = 50 as found by RTSE growth evolution studies. The depositing material fills the void space in the surface roughness layer of the underlying film. The protrusions in the surface roughness of the substrate film are coated with the depositing material, generating an interface layer associated with the growing film. Bruggeman effective medium approximation defines spectra in *ε* for these interfaces.

#### 3.3.1. Phonon Mode Variations in ZnO

**Figure 9:**
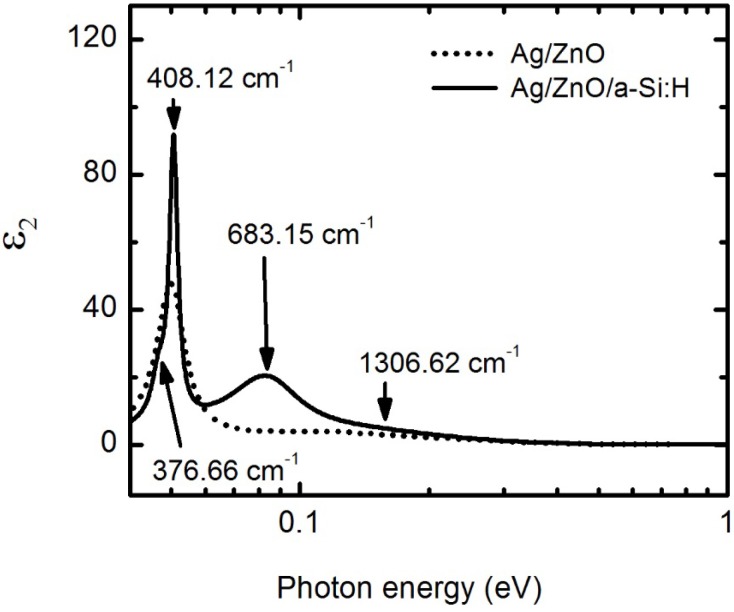
Comparison of lower energy features in *ε*_2_ as a function of photon energy for ZnO with (solid line) and without (dotted line) over-deposition of a-Si:H. Parameters describing the sample without and with over-deposition of are listed in Table 4 and Table 5, respectively.

Differences in *ε* for the ZnO are expected when over-coated with a-Si:H. PECVD of a-Si:H raises the temperature of ZnO to 200°C and exposes it to hydrogen in the plasma. There are many studies on the growth and various effects of annealing on the optical and structural properties of ZnO layers [65,66,67,68,69,70]. It is well known that the properties of ZnO layers are strongly affected by not only the deposition conditions but also the post-deposition annealing conditions or temperature treatments. Annealing has a large effect on the crystallinity of the layers in terms of grain size, residual strain, and the defect density as compared to as-deposited films. As noted in Table 5, the amplitude of the CPPB oscillators of ZnO over-coated with a-Si:H are fit to account for changes in *ε* occurring during PECVD of the a-Si:H layers. The increase in amplitude for higher energy absorption features in *ε* and the decrease in film thickness compared to the sample without a-Si:H coating at *T* = 200 °C indicate that the as-deposited ZnO film densifies and increases the degree of crystallinity after annealing at the a-Si:H deposition temperature. These variations are generally consistent with literature [69,70], in that the imaginary component of the optical response related to electronic transitions increases in amplitude and sharpens. However, Liu *et al.* reports a decrease in the real part of the complex index of refraction in the transparent region, which they attribute to void forming along with larger crystalline grains. In our samples, void formation is not observed optically, however the decrease in thickness implies that crystallite growth has occurred which coupled with the higher observed real part of *ε* indicates that this film is now overall more densely packed. The comparison of different phonon modes in ZnO with and without a-Si:H coating is shown in Figure 9. The characteristic TO modes with A_1_ and E_1_ symmetry at 0.0467 eV (376.66 cm^−1^) and 0.0506 eV (408.12 cm^−1^) are present and able to be resolved [51,52,53]. The vibrational mode at 0.0847 eV (683.15 cm^−1^) can be attributed to longitudinal optical (LO) mode with E_1_ symmetry [52]. The orientation of grains in the film could be a reason for shifting of modes to slightly higher or lower wavenumbers. The splitting of the peak observed at 404 cm^−1^ for the uncoated ZnO into two expected peaks at 377 and 408 cm^−1^ for ZnO over-coated with a-Si:H and appearance of the 683 cm^−1^ mode is likely due to grain size increases or a reduction in defect density from annealing at the a-Si:H deposition temperature of 200°C [68]. The increase in amplitude for *ε*_2_ of phonon mode at 408 cm^−1^ also supports the idea that grain restructuring and material densification occurs. The presence of an additional absorption mode at 1306.62 cm^−1^ (0.162 eV) can be associated with oxygen-hydrogen (O-H) bonds in the thin film, such as the formation of zinc hydroxide or absorbed water or stretching modes of hydrogen bonded to heavier elements like zinc [54]. The large broadening of this absorption peak could be due to the modification or damage to the ZnO as a result of exposure to hydrogen in the plasma.

#### 3.3.2. Chemical Bonding in a-Si:H

After ZnO deposition, a 278 Å thick *n*-layer was deposited onto a ZnO/Ag coated substrate with deposition conditions given in Table 1. The *n*-layer optical properties, as well as its *d_b_* and *d_s_*, were obtained from RTSE analysis. The final numerically inverted spectra in *ε* for the *n*-layer were fit to a Cody-Lorentz oscillator [71]. The Cody-Lorentz oscillator is described by:

(7) $$\varepsilon_{2}(E)=\{\begin{array}{ll} \frac{AE_{0}\Gamma E}{{\left(E^{2}-E_{0}^{2}\right)}^{2}+\Gamma^{2}E^{2}}\frac{{\left(E-E_{g}\right)}^{2}}{{\left(E-E_{g}\right)}^{2}+E_{p}^{2}} & E>E_{g}\\ 0 & E\leq E_{g} \end{array},$$

and

(8) $$\varepsilon_{1}\left(E\right)=\frac{2}{\pi }P\int_{0}^{\infty }\frac{\xi \;\varepsilon_{2}\left(\xi \right)}{\xi^{2}-E^{2}}d\xi$$

where *A* is the amplitude, *Γ* is the broadening, *E_0_* is the resonance energy, *E_g_* represents an absorption onset determined from a parabolic band constant dipole matrix element, and *E_p_* + *E_g_* represents the transition between Cody gap*-*like and Lorentz-like behavior. Analytical Kramers-Kronig transformation of *ε*_2_ yields *ε*_1_. Parameters describing *ε* for the *n*-layer at the deposition temperature *T* = 200 °C are *A* = 59 ± 2 eV, *Γ* = 2.12 ± 0.02 eV, *E_0_* = 3.99 ± 0.01 eV, *E_g_* = 1.58 ± 0.04 eV, and *E_p_* = 0.96 ± 0.09 eV.

**Figure 10:**
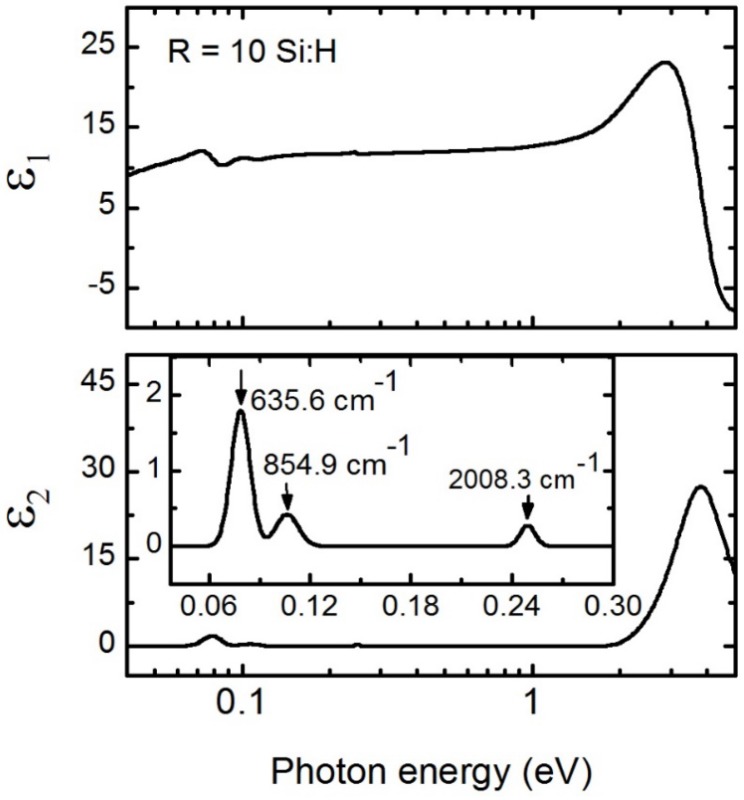
Spectra in *ε* (top panel, real part *ε*_1_; bottom panel, imaginary part *ε*_2_) extracted over a spectral range from 0.04 to 5 eV for 3621 ± 2 Å *R* = 10 a-Si:H films on BR over-coated with a *R* = 50 *n*-layer. The inset shows lower energy features in *ε*_2_ as a function of photon energy representing Si-H_n_ vibrational modes as modeled by Gaussian oscillators.

Figure 10 shows spectra in *ε* for the *R* = 10 a-Si:H intrinsic layer parameterized using a Cody-Lorentz oscillator at high energies and Gaussian oscillators to represent the IR vibrational modes. Each Gaussian oscillator [72] is described by:

(9) $$\varepsilon_{2}(E)=Ae^{-{\left(\frac{E-E_{n}}{\sigma }\right)}^{2}}-Ae^{-{\left(\frac{E+E_{n}}{\sigma }\right)}^{2}}$$

(10) $$\sigma =\frac{\Gamma }{2\sqrt{\ln\left(2\right)}}$$

where *A*, *Γ*, and *E_n_* represent amplitude, broadening, and resonance energy respectively, and *ε*_1_ is generated by Kramers-Kronig transformation of *ε*_2_ (Equation 8). Fit parameters are listed in Table 5. The Cody-Lorentz oscillator parameters for intrinsic a-Si:H were linked to a single fit parameter, *E_g_* from transmission and reflection spectroscopy, by linear relationships previously determined for PV device quality a-Si:H [71]. This technique minimizes the number of fit parameters allowing for extraction of physically realistic *ε*. Parameters describing spectra in *ε* for the underlying *n*-layer were extrapolated based on previously observed trends in the Cody-Lorentz oscillator parameters with temperature [73].

IR vibrational studies of a-Si:H have been useful in understanding the role of Si-H bonding in determining a-Si:H properties. High mobility and reactivity of hydrogen enables passivation of the electronic defect states in a-Si:H and relaxes the a-Si:H network to improve electronic and structural properties. IR-absorption studies have shown that hydrogen in a-Si:H is bonded as Si-H*_n_*, with *n* = 1, 2, and 3 [35,74]. IR features in *ε* for the intrinsic a-Si:H film are highlighted in the inset of Figure 10. Spectra in *ε* for a-Si:H in the *n-i-p* device configuration exhibited bending modes near 0.079 eV (635.6 cm^−1^) and a stretching monohydride (Si-H) mode around 0.249 eV (2008.3 cm^−1^). In addition to the expected Si-H modes, this a-Si:H sample exhibited an absorption mode centered around 0.106 eV (854.9 cm^−1^), which can be attributed to the bending or scissoring Si-H_2_ dihydride mode. The peak centered ~2100 cm^−1^ assigned to the dihydride (Si-H_2_) or clustered hydrogen is not observed. Although Si-H and Si-H_2_ bonding modes were previously resolved in ellipsometric measurements for other samples [75], we can resolve only the Si-H peak here possibly due to a much lower amplitude of the Si-H_2_ peak or reduced sensitivity to that feature in this particular sample. In addition to mode deconvolution in *ε*, the Si-H_2_ mode is also not observed in the extinction coefficient, *k*, or the absorption coefficient, *α*, obtained from *ε*. The absence of that peak usually confirms the presence of ordered dense Si:H material [76,77]. The amplitude of *ε*_1_ and the relatively high amplitude of the near IR to UV absorption feature in *ε*_2_ indicate that this is dense material and suitable for PV devices.

## 4. Summary and Conclusions

RTSE and IR-SE have been demonstrated as a useful metrology technique for characterization of PECVD Si:H layers and components of the BR structure used in *n*-*i*-*p* a-Si:H solar cells. Growth evolution diagrams were developed for *n*-type, intrinsic, and *p*-type Si:H to identify the regions of optimized protocrystalline a-Si:H material for the respective thicknesses used in the solar cell configuration. IR to UV *ex situ* SE measurements and analysis were used to determine spectra in *ε* for Ag, ZnO, the ZnO + Ag interface, and protocrystalline intrinsic a-Si:H in the device configuration. Free carrier absorption in Ag and the ZnO + Ag interface, the particle plasmon feature in the ZnO/Ag interface, and four IR phonon modes in ZnO were identified. Si-H*_n_* bonding modes were identified in *ε* obtained from intrinsic a-Si:H prepared on a *n*-type a-Si:H coated BR. IR-SE has been demonstrated to be sensitive to bonding characteristics of a-Si:H layers in the PV device configuration. Overall, the results and analysis procedures developed here are applicable to more directly relating film properties, as obtained by non-destructive measurements in the PV device configuration, with variations in device performance.

## Figures and Tables

**Table 1:** Deposition conditions for the individual layers in the a-Si:H *n*-*i*-*p* solar cell configuration deposited on 6" × 6" borosilicate glass substrates. The 5% dopant gas in H_2_ is by volume. Cr, Ag, and ZnO were sputtered at room temperature (RT).

Layer	Substrate Temperature T (°C)	Pressure p (mTorr)	Radio Frequency (rf) Power P (W/cm^2^)	Gas Flow (sccm)				
				Ar	SiH_4_	5% PH_3_ or B_2_H_6_ in H_2_	H_2_	*R* = [H_2_]/[SiH_4_]
Cr	RT	5	0.92	10	-	-	-	-
Ag	RT	5	0.92	10	-	-	-	-
ZnO	RT	5	0.92	10	-	-	-	-
*n*	200	1500	0.032	-	2	0.5 PH_3_	40–160	20–80
*i*	200	800	0.04	-	5	-	50–250	10–50
*p*	100	1500	0.066	-	2	0.5 B_2_H_6_	100–400	50–200

**Table 2:** Parameters describing complex dielectric function (*ε* = *ε*_1_ + i*ε*_2_) and structure for a semi-infinite Ag film on a borosilicate glass over coated by Cr before ZnO deposition. Experimental ellipsometric spectra were collected *in situ* after deposition at room temperature in the spectral range from 0.734 to 5.88 eV and fit using least square regression analysis with an unweighted estimator error function, σ = 5 × 10^−3^. For bulk Ag, the parameterization of *ε* consisted of a Drude oscillator, two oscillators assuming critical point parabolic bands (CPPB), and a constant additive term to *ε*_1_ denoted *ε*_∞_. Spectra in *ε* for the 30 ± 2 Å surface roughness layer were parameterized with two Lorentz oscillators and *ε*_∞_ = 1.

**Ag Surface Roughness**					
**Oscillator**	**A (Unitless)**	**Γ** **(eV)**	**E_0_ (eV)**	-	-
Lorentz	4.2 ± 0.2	2.5 ± 0.1	5.17 ± 0.02	-	-
Lorentz	1.0 ± 0.3	0.06 ± 0.03	3.61 ± 0.01	-	-
**Bulk Ag**					
**Oscillator**	**A (Unitless)**	**Γ** **(eV)**	**E_n_ (eV)**	**Ө (degrees)**	**µ**
CPPB	5.29 ± 0.09	0.70 ± 0.03	3.845 ± 0.008	−180.306 ± 0.002	0.5
CPPB	10.39 ± 0.07	0.87 ± 0.01	4.025 ± 0.001	−7.0 ± 0.4	0.5
Drude	**ρ** **(Ωcm)**		**τ** **(fs)**		
Constant additive term to ε_1_	3.02 ± 0.03 × 10^−6^		16.7 ± 0.1		
ε_∞_	1.632 ± 0.008				

**Table 3:** Parameters describing *ε* and structure for a ZnO film deposited on Ag and the ZnO + Ag interface formed. Experimental ellipsometric spectra were collected *in situ* after deposition at room temperature in the spectral range from 0.734 to 5.0 eV and fit using least squares regression analysis with an unweighted estimator error function, σ = 7 × 10^−3^. Parameters describing *ε* for Ag were fixed from Table 2. For ZnO, the parameterization of *ε* consisted of two CPPB oscillators, a Sellmeier oscillator, and *ε*_∞_. For the ZnO + Ag interface, the parameterization of *ε* consisted of a Drude oscillator, a Lorentz oscillator, and *ε*_∞_.

Layer	Oscillators			
ZnO *d_b_* = 3060 ± 3 Å *d_s_* = 80 ± 1 Å	CPPB (µ = 0.5)		ε_∞_ = 2.27 ± 0.01	
	A (Unitless)	Γ (eV)	E_n_ (eV)	Ө (degrees)
	2.63 ± 0.02	0.199 ± 0.002	3.363 ± 0.001	−20.1 ± 0.5
	1.41 ± 0.02	3.83 ± 0.08	4.36 ± 0.03	0 (fixed)
	Sellmeier			
	A (eV^2^)	Γ (eV)		E_n_ (eV)
	0.080 ± 0.002	-		0
ZnO/Ag Interface = 108 ± 11 Å	Lorentz		ε_∞_ = 1	
	A (Unitless)	Γ (eV)		E_0_ (eV)
	2.8 ± 0.2	0.57 ± 0.05		2.83 ± 0.01
	Drude			
	ρ (Ω cm)		τ (fs)	
	3.7 ± 0.5 x10^−5^		2.7 ± 0.3	

**Table 4:** Parameters describing *ε* and structure for a ZnO film deposited in a ZnO/Ag back reflector (BR). Experimental ellipsometric spectra were collected *ex situ* using near infrared to ultraviolet (0.734 to 5.0 eV) and infrared (0.04 to 0.734 eV) spectral range instruments and fit jointly using least squares regression analysis with an unweighted estimator error function, σ = 8 x 10^−3^. Parameters describing *ε* for Ag and the ZnO + Ag interface were fixed from Table 2 and Table 3, respectively. The ZnO bulk layer thickness was allowed to vary separately for each set of ellipsometric spectra; all other parameters are common to both analyses. For ZnO, the parameterization of *ε* consisted of two CPPB oscillators, three Lorentz oscillators, and *ε*_∞_.

Layer	Oscillators			
ZnO *d_b_* (Near IR to UV) = 2996 ± 2 Å *d_b_* (IR) = 3025 ± 2 Å *d_s_* = 84 ± 1 Å	CPPB (µ = 0.5)		ε_∞_ = 2.43 ± 0.01	
	A (Unitless)	Γ (eV)	E_n_ (eV)	Ө (degrees)
	2.82 ± 0.02	0.209 ± 0.002	3.364 ± 0.001	−20.8 ± 0.4
	1.23 ± 0.02	3.95 ± 0.03	3.94 ± 0.02	0
	Lorentz			
	0.75 ± 0.05	0.196 ± 0.005	0.264 ± 0.002	-
	3.17 ± 0.03	0.169 ± 0.007	0.134 ± 0.001	-
	46 ± 2	0.0093 ± 0.0004	0.0501 ± 0.0002	-

**Table 5:** Parameters describing *ε* and structure for a ZnO/Ag BR coated with *n*-type and intrinsic a-Si:H. Experimental ellipsometric spectra were collected *ex situ* using near infrared to ultraviolet (0.734 to 5.0 eV) and infrared (0.04 to 0.734 eV) spectral range instruments and fit jointly using least squares regression analysis with an unweighted estimator error function, σ = 11 × 10^−3^. Parameters describing *ε* for Ag and the ZnO + Ag interface were fixed from Table 2 and Table 3, respectively. Parameters describing *ε* for the *n*-layer were determined from RTSE analysis of data collected at *T* = 200 °C, parameterized by a Cody-Lorentz oscillator, and then parameter values extrapolated to room temperature. The ZnO and intrinsic a-Si:H bulk layer thicknesses were allowed to vary separately for each set of spectra; all other parameters are common to both analyses. For ZnO, the parameterization of *ε* consisted of two CPPB oscillators with all parameters except the amplitudes fixed to the values in Table 4, four Lorentz oscillators, and *ε*_∞_. For a-Si:H layers, the parameterization of *ε* was based on a Cody-Lorentz oscillator and *ε*_∞_. A Sellmeier oscillator and three Gaussian oscillators were added to the parameterization of *ε* for intrinsic a-Si:H.

Layer	Oscillators				
*i*-type a-Si:H *d_b_* (Near IR to UV) = 3623 ± 1 Å *d_b_* (IR) = 3619 ± 2 Å *d_s_* = 29.0 ± 0.3 Å	*i*-type a-Si:H		Cody-Lorentz E_g_ (T&R) = 1.780 ± 0.001; ε_∞_ = 1.50 ± 0.01		
	Gaussian				
	A (Unitless)			Γ (eV)	E_0_ (eV)
	1.732 ± 0.06			0.013 ± 0.001	0.079 ± 0.001
	0.28 ± 0.01			0.010 ± 0.001	0.249 ± 0.001
	0.41 ± 0.04			0.016 ± 0.002	0.106 ± 0.001
	Sellmeier				
	0.0050 ± 0.0002 eV^2^			-	0
*i*- a-Si:H/*n*-type a-Si:H Interface = 30 ± 1 Å *n*-layer *d_b_* = 278 ± 1 Å *n*-type a-Si:H/ZnO Interface = 84 Å	*n*-type a-Si:H		Cody-Lorentz; ε_∞_ = 1		
	A (eV)	Γ(eV)	E_0_ (eV)	E_g_ (eV)	E_p_ (eV)
	62	2.01	3.99	1.65	1.05
ZnO *d_b_* (Near IR to UV) = 2763 ± 3 Å *d_b_* (IR) = 2738 ± 5 Å	ZnO		CPPB (µ = 0.5) ε_∞_ = 1.91 ± 0.02		
	A		Γ (eV)	E_n_ (eV)	Ө
	4.04 ± 0.05		0.209	3.364	−20.8
	1.31 ± 0.02		3.95	3.94	0
	Lorentz				
	A (Unitless)			Γ (eV)	E_0_ (eV)
	3.89 ± 0.1			0.233 ± 0.001	0.162 ± 0.002
	82.0 ± 4.0			0.0030 ± 0.0003	0.0506 ± 0.0001
	16.4 ± 0.4			0.039 ± 0.002	0.085 ± 0.001
	13.0 ± 3.0			0.004 ± 0.002	0.047 ± 0.001
